# Tunisian Native *Mentha pulegium* L. Extracts: Phytochemical Composition and Biological Activities

**DOI:** 10.3390/molecules27010314

**Published:** 2022-01-05

**Authors:** Jed Jebali, Hanene Ghazghazi, Chedia Aouadhi, Ines ELBini-Dhouib, Ridha Ben Salem, Najet Srairi-Abid, Naziha Marrakchi, Ghayth Rigane

**Affiliations:** 1 Laboratory of Biomolecules, Venoms and Theranostic Applications, LR20IPT01, Institut Pasteur of Tunis, University of Tunis El Manar, Tunis 1002, Tunisia; inesbini@yahoo.fr (I.E.-D.); najetsrairi@yahoo.fr (N.S.-A.); marrakchi_naziha@yahoo.fr (N.M.); 2 Laboratory of Management and Valorization of Forest Resources, National Research Institute of Rural Engineering, Water and Forestry (INRGREF), University of Carthage, Tunis 1004, Tunisia; hanene8116@yahoo.fr; 3 Laboratoire d’Epidémiologie et Microbiologie Vétérinaire, Groupes de Bactériologie et Développement Biotechnologique, Institut Pasteur de Tunis, Université de Tunis El Manar, 13, Place Pasteur, B.P. 74, Tunis 1002, Tunisia; chediaaouadhi@yahoo.fr; 4 Laboratory of Organic Chemistry LR17ES08, Faculty of Sciences of Sfax, University of Sfax, B.P. 1171, Sfax 3038, Tunisia; Ridha.bensalem@fss.rnu.tn (R.B.S.); gaith.rigane@yahoo.fr (G.R.); 5 Medicine School of Tunis, 15 Djebel Lakhdhar, Street La Rabta, University of Tunis El Manar, Tunis 1007, Tunisia; 6 Chemistry-Physics Department, Faculty of Sciences and Technology of Sidi Bouzid, University of Kairouan, B.P. 380, Sidi Bouzid 9100, Tunisia

**Keywords:** medicinal plant, polyphenols, antioxidant, antiproliferative and antibacterial activities

## Abstract

Mint species (*Lamiaceae* family) have been used as traditional remedies for the treatment of several diseases. In this work, we aimed to characterize the biological activities of the total phenolic and flavonoid contents of *Mentha pulegium* L. extracts collected from two different regions of Tunisia. The highest amounts of total phenols (74.45 ± 0.01 mg GAE/g DW), flavonoids (28.87 ± 0.02 mg RE/g DW), and condensed tannins (4.35 ± 0.02 mg CE/g DW) were found in the Bizerte locality. Methanolic leaf extracts were subjected to HPLC-UV analysis in order to identify and quantify the phenolic composition. This technique allowed us to identify seven phenolic compounds: two phenolic acids and five flavonoid compounds, such as eriocitrin, hesperidin, narirutin, luteolin, and isorhoifolin, which were found in both extracts with significant differences between samples collected from the different regions (*p* < 0.05). Furthermore, our results showed that the methanolic extract from leaves collected from Bizerte had the highest antioxidant activities (DPPH IC_50_ value of 16.31 μg/mL and 570.08 μmol Fe^2+^/g, respectively). Both extracts showed high radical-scavenging activity as well as significant antimicrobial activity against eight tested bacteria. The highest antimicrobial activities were observed against Gram-positive bacteria with inhibition zone diameters and MIC values ranging between 19 and 32 mm and 40 and 160 µg/mL, respectively. Interestingly, at 10 μg/mL, the extract had a significant effect on cell proliferation of U87 human glioblastoma cells. These findings open perspectives for the use of *Mentha pulegium* L. extract in green pharmacy, alternative/complementary medicine, and natural preventive therapies for the development of effective antioxidant, antibacterial, and/or antitumoral drugs.

## 1. Introduction

The Mentha plant grows all year round, mainly in the Mediterranean area, where it forms a dominant part of the vegetation [1,2]. The genus of Mentha is among the major genera belonging to the Lamiaceae family and comprises more than 60 species according to the latest taxonomic ranking. In Tunisia, the Mentha genus is represented mainly by the *M. pulegium* L., *M.* *rotundifolia* L., *M. longifolia* (L.) Huds., *M. spicata* L. (*M. viridis* L.), and *M. aquatica* L. species [3,4,5]. Mint species have been shown to present several virtues and have been used for different purposes, such as in culinary uses to improve aroma and flavor, as well as in cosmetics [3,4,5]. Mint species have also been used for medicinal aims, such as in infusions or tinctures for the treatment of intestinal colic, liver disorders, gastritis, and jaundice, as well as for headaches and migraine [6]. Mint extracts were found to contain a wealth of compounds, collectively named terpenoids and polyphenols, which include phenolic acids, flavones, and flavanols, in their free forms or as glycoconjugates [7]. Similar components were also found in the leaves and flowers of small wild plants growing in Saudi Arabia named *Lantana camara*, *Anvillea garcinii*, and *Strychnos nux-vomica* [8,9,10]. In vitro studies showed that mint extracts from the Tunisian genus exhibited important antioxidant, antiviral, antiallergenic, and antimicrobial activities [11,12,13]. In addition, previous studies have demonstrated the potent antitumor activities of the Mentha species [14,15] as an effective chemopreventive agent [16]. For instance, *Mentha aquatica* showed a selective antiproliferative activity on MCF-7 breast cancer cell lines [17]. On the other hand, several researchers have determined the biological activities and chemical composition of Mentha extracts; however, according to our knowledge, the antibacterial and antioxidant activities of the Tunisian Mentha species have been scarcely studied, and there are no available data on the activity of this genus against glioblastoma cancer cell lines. Therefore, the aim of this study is to assess the composition of the Tunisian *Mentha pulegium* L., analyze its variability from two geographical zones belonging to different bio-climates (Bizerte and Kef), and evaluate its biological effects. The assessment of variation is crucial for identifying interesting chemotypes and defining appropriate strategies for ethnopharmacological uses. Furthermore, we aimed to evaluate, for the first time, the potential antitumoral effect of *Mentha pulegium* L. extracts against U87 cells derived from human glioblastoma, the most deadly brain cancer.

## 2. Results and Discussion

### 2.1. Determination of Yields, Phenols, Flavonoids, and Condensed Tannin Contents

The results of the maceration extraction yielded 22 ± 0.23%. Then, the content of total phenols, flavonoids, and condensed tannin was determined by spectrophotometric methods. As shown in Table 1, the secondary metabolite contents in the tested methanolic extracts varied according to the site of sampling. In fact, a higher condensed tannin and flavonoid content was observed in the leaves collected from the Bizerte locality (*p* < 0.05).

The contents of the polyphenols found in the *Mentha pulegium* L. methanolic extracts were overall more significant than those described in the literature for the same species of the genus *Mentha* such as *M. pulegium* L. In fact, Hajlaoui et al. (2008) demonstrated that the content of polyphenols in an *M. pulegium* L. extract was 37.4 mg GAE/g DW [18]. In addition, Karray-Bouraoui et al. (2010) reported wide ranges varying from 20.1 to 56.6 mg GAE/g DW for polyphenols in the methanolic extract of *M. pulegium* L. [19].

### 2.2. Determination of Phenol Contents

**Figure 1:**
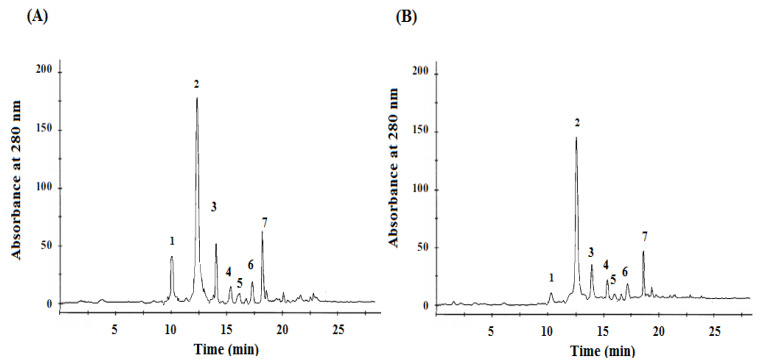
HPLC chromatograms of *Mentha pulegium* L. methanolic extracts (Bizerte (**A**) and Kef (**B**)). Peaks: 1, caffeic acid; 2, eriocitrin; 3, isorhoifolin; 4, luteolin; 5, narirutin; 6, hesperidin; and 7, rosmarinic acid detected at 280 nm.

The identification and quantification of phenolic compounds were determined by HPLC (Figure 1). An important difference between the phenolic phytochemical profile analysis of the *Mentha pulegium* L. methanolic extract obtained from two different origins was observed (Figure 1). The detected compounds were classified as phenolic acids: gallic and rosmarinic acids, and flavonoids such as: eriocitrin, isorhoifolin, hesperidin, luteolin, and narirutin. As summarized in Table 2, there were significant differences between the two *Mentha pulegium* L. origins (*p* < 0.05). According to this table, we could conclude that eriocitrin is the major flavonoid compound quantified in the *Mentha pulegium* L. leaves (20.1–25.3 mg/g of DW) (*p* < 0.05), followed by isorhoifolin (1.2–2.5 mg/g of DW) (*p* < 0.05), and hesperidin (0.5–0.75 mg/g of DW) (*p* < 0.05). On the other hand, rosmarinic acid was the major phenolic acid present in the *Mentha pulegium* L. leaves.

By comparing our results with those of the literature, a similar phenolic composition was found in plants cultivated in the south of India [20] and in the dried leaves of peppermint (*Mentha piperita* L.) [21]. Moreover, our plant was found to be composed by the same phenolic compounds as those of *Menthae piperitae folium*, as reported recently by Bodalska et al. (2019) [22].

Even the most commonly identified compounds were present in both of the investigated samples; significant quantitative variations were recorded for the polyphenol components in both of the studied localities. Indeed, the amount of total polyphenols was higher in Bizerte than in Kef region. Our results are in agreement with previous reports that noticed the presence of different chemotypes for the species *M. rotundifolia* L. growing in various parts of Tunisia [23] or other parts of the world [24,25].

In the present study, the variation in phenolic compounds observed for the two studied localities was strongly related to abiotic factors such as the samples’ climate-specific regions of provenance and geographical factors such as altitude and soil type [26].

### 2.3. Antioxidant Activity

The antioxidant properties of plant extracts, in foods and biological systems, can be evaluated using various in vitro assays. These assays can be divided in two groups: (a) those evaluating lipid peroxidation and (b) those that measure free radical-scavenging ability [27]. In our study, the antioxidant activity of *Mentha pulegium* L. extracts was measured with different methods to evaluate the free radical-scavenging potentials (DPPH) and metal (Fe^2+^ radicals) chelation potential. Our results (Table 3) confirmed the positive relationship between the antioxidant potential of *Mentha pulegium* L. plant and their polyphenolic compounds. In fact, the lowest DPPH IC_50_ value was correlated with the highest antioxidant activity. In contrast, for the Fe^2+^ assay, a higher value indicated higher antioxidant activity.

According to the obtained results (Table 3), the DPPH and Fe^2+^ radical-scavenging potentials of the Bizerte methanolic extract (IC_50_ = 16.31 µg/mL and 570.08 μmol Fe^2+^/g, respectively (*p* < 0.05)) were higher than those from the Kef site (IC_50_ = 19.08 µg/mL and 481.01 μmol Fe^2+^/g, respectively (*p* < 0.05)). Such activities may be due to the phenols present in these extracts as reported in Table 2. Indeed, phenolic compounds have been reported to have multiple biological effects, including antioxidant activity. They may act as free radical scavengers or prevent their formation. The overall antioxidant potential of the two plants seems to be correlated to the presence of rosmarinic acid, caffeic acid, and eriocitrin [28,29]. When compared to same and other Mentha species, *Mentha pulegium* L. methanolic extracts showed stronger DPPH radical-scavenging activity than those described in the literature. Indeed, Ghazghazi et al. (2013) and Hajlaoui et al. (2010) showed that the IC_50_ values of *M. pulegium* L. and *M. longifolia* L. methanolic extract were of 56 µg/mL and 20 µg/mL, respectively [5,30].

### 2.4. Antibacterial Activity

The antibacterial activity of methanolic extracts from the leaves of the species *Mentha pulegium* L. collected in two Tunisian regions against eight Gram-positive and Gram-negative bacteria was assessed by determining the inhibition zones (Table 4 and Table 5). The results, summarized in Table 4, show that both extracts had good antibacterial activity against all the tested pathogens. As represented by the MIC and MBC values, we remarked that the effect varied according to the microorganism species. Indeed, based on the results reported in Table 4 and Table 5, it is interesting to point out that the Gram-positive bacteria were more susceptible to the tested extract than the Gram-negative ones. The highest inhibition zone diameters were obtained for Gram-positive bacteria, ranging from 22 mm (*Clostridium tetani*) (*p* < 0.05) to 33 mm (*S. aureus*) (*p* < 0.05). The lowest value was observed for Gram-negative bacteria such as *Klebsiella pneumoniae* (IZ = 19 mm) (*p* < 0.05). Our results correlated with those of previous studies [31,32,33,34,35,36], which could be explained by the presence of hydrophobic lipopolysaccharide in the outer membrane providing protection against different agents [37,38].

The results of the bacteriostatic and bactericidal activities of the methanol extracts against the tested bacteria are listed in Table 5 and confirm the disc diffusion results. In fact, the two tested extracts showed important antibacterial activity with the MIC and the MBC values in the ranges of 40–80 µg/mL and 80–160 µg/mL for the Bizerte locality and 80–160 µg/mL and 160–320 µg/mL for the Kef locality, respectively. The difference in the antibacterial effects of the extracts from the two regions is worth noting. It is clear that the extract of the plant from the Bizerte region is more active than that from Kef region. In their study, Ghazghazi et al. (2013) and Gulluce et al. (2007) showed that the methanol extracts of the aerial parts of *M. pulegium* L. and *M. longifolia* ssp. plants had no antimicrobial activities [5,39], indicating that our *M. pulegium* species has a more interesting effect.

Thus, as for the antioxidant capacity, we observed differences in the antibacterial activities of the methanolic extract of *Mentha* *pulegium* L. leaves collected from two provinces. Such a difference may be related to the variation in the phenol and flavonoid contents as reported in Table 1 and Table 2. The strong antibacterial properties can be attributed to their phenolic compounds, namely, rosmarinic acid, luteolin, and caffeic acid. Indeed, these compounds are known for their antimicrobial and antiviral activities and strong antioxidant and antitumor action [25]. The presence of hydroxyl groups and their relative position in the phenolic ring is probably responsible for the strong antibacterial activity because of the ability of these substances to bind to bacterial membranes. In fact, phenolic compounds from mint could destroy the permeability barrier of bacteria and cause the release of intracellular constituents such as ribose and sodium glutamate [40]. Moreover, they interfere with electron transport, nutrient uptake, protein and nucleic acid synthesis, and enzyme activity, leading to the inhibition of bacterial growth [40].

### 2.5. Cytotoxicity and Antiproliferative Activities

**Figure 2:**
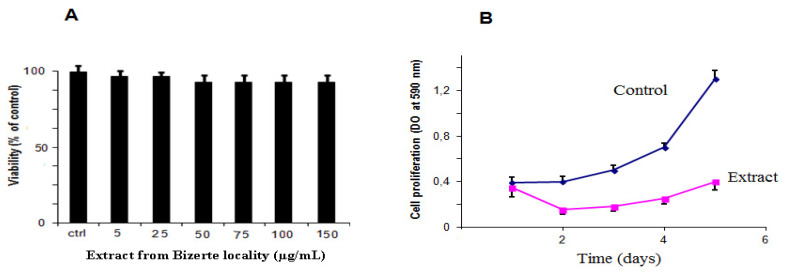
Effect of methanolic extract of *Mentha pulegium* L. from the Bizerte locality on U87 cells: (**A**) Cells were treated with the extract at the indicated concentrations for 24 h, and cell viability was assessed by MTT assay. The cell viability of the vehicle control cells was assumed as 100%. (**B**) The methanolic extract from the Bizerte locality inhibited U87 cell proliferation. U87 cells were cultured in MEM containing 10% FCS for the indicated periods of time in the absence (control) or in the presence of 10 µg/mL of extract. At daily intervals, U87 cells were fixed with 3.7% formaldehyde, stained with a solution of 0.1% crystal violet/MetOH 20%, and lysed with 1% SDS. Absorbance was then measured at 590 nm. All data represent the mean ± SEM of three separate experiments performed in triplicate (*p* < 0.05).

The originality of this work consists of demonstrating a new biological activity for the methanolic extracts of *Mentha* *pulegium* L. Indeed, the antiproliferative activity against human glioma cells has not yet been reported. Since the best activity was obtained with the Bizerte plant extract, we chose it to investigate its effect on U87 human glioblastoma cells. Firstly, we assessed the effect of the methanolic extract on cell viability using an MTT assay [41]. As shown in Figure 2A, methanolic extract (from 5 to 150 µg/mL) was unable to affect the viability of U87 cells after 24 h of treatment. Interestingly, our results demonstrated that the methanolic extract had a significant effect on cell proliferation when applied at 10 μg/mL. The inhibition of proliferation was observed from the second day and reached 75% on the fifth day (*p* < 0.05), compared to untreated cells (Figure 2B).

This interesting observed effect is probably due to the different polyphenol compounds of the *Mentha pulegium* L. methanolic extract. Indeed, polyphenolic compounds have been previously reported for their antitumor action [42,43,44]. For instance, Hossan et al. reported anticancer activities of rosmarinic acid and discussed its therapeutic potential against a variety of cancers including colon and skin cancers [42]. In addition, caffeic acid, an active component of propolis extract, specifically inhibits NF-κB and exhibits antioxidant, anti-inflammatory, and antiproliferative properties in SK-MEL-28 and PC-3 cells [44].

## 3. Conclusions

In summary, *Mentha pulegium* L. plant extracts from the northwestern part of Tunisia (Bizerte and Kef), rich in phenolic and flavonoid compounds, have been studied here for the first time. The plant extracts showed an important antioxidant activity as well as an antibacterial agent. Interestingly, this plant showed antiproliferative activity against U87 human glioblastoma cells. All the results indicate that the high content of total phenols in the methanolic extracts might be responsible for important biological activities. Taken together, Tunisian *Mentha pulegium* L. is a promising medicinal plant for the development of a new generation of dietary supplements that can be used as antibacterial, antitumoral, and antioxidant agents.

## 4. Materials and Methods

### 4.1. U78 Cell Lines and Chemicals

The U78 cells were cultured in MEM containing 10% fetal bovine serum (FBS; Sigma, St. Louis, MS, USA). Cells were maintained in a humidified atmosphere of 5% CO_2_ at 37 °C. Before the cell proliferation assays, U87 cells were trypsinized and seeded in 96-well round-bottomed tissue culture plates. The MTT (3-(4, 5-Dimethylthiazol-2-yl)-2,5- Diphenyltetrazolium Bromide), penicillin and streptomycin mixture, l-glutamine (200 mM), and phosphate buffer saline (PBS) were from GIBCO-BCL. All plastic wares for cell culture were obtained from Techno Plastic Products AG, Trasadingen (Switzerland).

### 4.2. Plant Material

The leaves of *Mentha pulegium* L. (*Mentha pulegium*) were collected in January, separately, from two regions situated in the northern part of Tunisia: Bizerte (N: 36.7271°, E: 9.1880°, sub-humid) and Kef (N: 36.1110°, E: 8.4200°, semi-arid). The leaves were dried at room temperature, and the botanical identity of the collected samples (Family, Genus, and Species) was determined by Professor M. Sadok Bouzid, a botanist in the Faculty of Sciences of Tunis. A voucher specimen was deposited in the laboratory.

### 4.3. Extraction of Plant Material

Only healthy leaves were harvested and immediately transported to our laboratory. After drying at ambient temperature (~20 °C), the leaves were all ground using an electric mill (Retsch Muhle, Grindomix, GM200, Kurt Retsch GmbH & Co. KG, Haan, Germany), at 10,000 rpm/min, using a 0.5 mm mesh screen to improve contact with the solvent [45]. Briefly, 15 g of the obtained samples was extracted by stirring with 100 mL methanol/water solvent (80:20 *v*/*v*) for 24 h in a bath water shaker maintained at 30 °C in the dark. The obtained extracts were filtered through a filter paper and then centrifugated at 2500 rpm for 10 min. Then, the filtrate was evaporated under a vacuum. The dried crude concentrated extracts were weighed to calculate the yield. Five extraction replicates were performed for each sample and stored in a refrigerator at 4 °C until used for analyses.

### 4.4. Determination of Total Flavonoid Contents

Total flavonoid contents were determined by the aluminum trichloride method [38]. Briefly, 1.5 mL of 2% aluminum trichloride (AlCl_3_) in methanol was added to 1.5 mL of extract, and the volume was adjusted to 26 mL with methanol. The mixture was incubated for 45 min, and the absorbance was measured by a spectrophotometer (Tokyo, Japan) at 420 nm [46]. The results are given as rutin equivalents per gram dry weight (mg RE/g DW).

### 4.5. Condensed Tannin Content

Condensed tannins of plant extract were determined as previously described by Sun et al. [47], and they were expressed as mg catechin equivalents per gram dry weight (mg CE/g DW) through the calibration curve with catechin. Briefly, 50 μL of the methanolic extract was mixed with 3 mL of 4% methanol vanillin solution and 1.5 mL of H_2_SO_4_. After incubation for 15 min, the absorbance of the tested extract was measured at 500 nm.

### 4.6. Phenolic Compounds Content

Total phenolic contents were determined using the Folin–Ciocalteu method as described by Slinkard et al. (1977) [48]. The calibration curve was prepared with gallic acid, and the result was expressed as mg gallic acid equivalents per gram dry weight (mg GAE/g DW). The phenolic composition of *Mentha pulegium* L. was quantitatively determined by high-performance liquid chromatography (HPLC) as described in [21]. HPLC analysis was performed using an Agilent 1100 HPLC system including an autosampler, a vacuum degasser, a thermostatted column compartment, a quaternary pump, and a diode array detector (column temperature 40 °C) (Agilent 1100). The column was a Beta Basic-18 C-18 (5 µm, 25 cm × 4.6 mm i.d.) (Thermo Hypersil, UK). An Agilent LC-3D ChemStation for LC systems was used, and chromatograms were obtained at 280 nm. The flow rate was 1 mL/min, and the injection volume was 20 µL. Individual phenols of the *Mentha pulegium* L. extracts were expressed as mg·g^−1^. The eluents were 1.5% formic acid in acetonitrile (A) and 1.5% formic acid in water (B), and the gradient time was s 25 min gradient of 15% to 45% of solvent B. Individual phenols were quantified by a three-point regression curve on the basis of standards obtained from commercial suppliers with the exception of eriocitrin, hesperidin, narirutin, luteolin, rosmarinic acid, caffeic acid, and isorhoifolin.

### 4.7. Antioxidant Activity Evaluation

#### 4.7.1. Free Radical-Scavenging Activity (DPPH)

The DPPH radical-scavenging capacity was measured according to Hanato et al. (1988) [49]. Briefly, 1 mL of each extract (at different concentrations in methanol) was mixed with 0.5 mL of 0.2 mM DPPH methanolic solution. The reaction was allowed to stand at room temperature in the dark for 30 min, and the absorbance was recorded at 517 nm. The scavenging activity was estimated using the following equation: scavenging effect (%) = [100 × (Ac − A_S_/A_c_)], where Ac is the absorbance of the control reaction (containing all reagents except the plant extract), and A_S_ is the absorbance of the tested sample. The IC_50_ is the concentration of extract that could scavenge 50% of the DPPH radicals.

#### 4.7.2. Ferric-Reducing Power (FRAP) Assay

The FRAP reagent was freshly prepared by mixing acetate buffer (300 mM, pH 3.6), TPTZ solution (10 mM TPTZ in 40 mM HCl), and FeCl_3_-6H_2_O (20 mM) in a ratio of 10:1:1 [50]. To perform the assay, 900 μL of FRAP reagent, 90 μL distilled water, and 30 μL of plant extract were mixed and incubated at 37 °C for 15 min. The absorbance was measured at 595 nm using a FRAP working solution as a blank. The antioxidant potential of the samples was determined from a standard curve plotted using the FeSO_4_·7H_2_O linear regression. The results were expressed as µmol Fe^2+^ equivalents/g methanolic extract.

### 4.8. Antimicrobial Activity

The eight human pathogenic bacteria employed in this study were *Streptococcus aureus, Bacillus subtilis, Clostridium tetani, Enterococcus, Echerichia coli, Klebsiella pneumoniae, Shigella boydii*, and *Vibrio cholera*. These were obtained from clinical isolates at the Microbiology Department of Institute Pasteur of Tunisia. The identification of all these strains was confirmed by conventional procedures (cultural characterization and API system, Bio Merieux, Marcy-l'Étoile, France). The used bacteria were recovered by overnight growth at 37 °C in Mueller–Hinton broth at pH 7.4.

#### 4.8.1. Disc Diffusion Method

Preliminary screening for the antimicrobial activity of *Mentha pulegium* L. methanolic extracts was performed by a disc diffusion assay against Gram-positive and Gram-negative bacteria [51]. Briefly, 100 μL of bacterial suspension (10^8^ CFU/mL) were spread on Petri plates containing a Mueller–Hinton medium. The paper discs (6 mm in diameter) were separately impregnated with 15 μL of the different concentrations (0.1, 0.5, 2 mg/mL) of each methanolic extract in DMSO then placed on the agar, which was pre-inoculated with the selected microorganisms. Discs containing Gentamicin (15 µg/disc) were used as a positive control, and those without samples were used as a negative control. Plates were kept at 4 °C for 1 h. The inoculated plates were incubated for 24 h at 37 °C. Antimicrobial activity was assessed by measuring the diameter of the growth-inhibition zone in millimeters (including a disc diameter of 6 mm) for the test organisms as compared to the controls.

#### 4.8.2. Determination of MIC and MBC

The minimum inhibitory concentration (MIC) and minimum bactericidal concentration (MBC) of the *Mentha pulegium* L. methanolic extracts were determined using the agar dilution method as described by Ghazghazi et al. (2013) [5]. Briefly, Petri plates of Mueller–Hinton agar containing various concentrations of methanolic extract (20, 40, 80, 160, and 320 µg/mL) were inoculated with each tested strain. Each working culture (2 × 10^7^ CFU/mL) was diluted to obtain 10^4^ and 10^5^ in peptone water (0.1% *w*/*v*), and 50 µL of each diluted culture was individually spread on the surface of the solidified agar plates. The positive control consisted of Mueller–Hinton agar without methanolic extracts, inoculated with the diluted medium culture. All plates were incubated for 24 h at 37 °C, then evaluated for the presence or the absence of colonies. For each treatment, the absence of colonies on all the plates tested was considered an inhibitory effect. The lowest concentration of methanol extracts required to completely inhibit the growth of the tested microorganism was designated as the MIC.

### 4.9. Antitumoral Activity

#### 4.9.1. Cell Viability Assay

The viability assay was performed according to the manufacturer’s instructions (Promega, Madison, WI, USA). After starvation, U87 cells were harvested and treated with the methanolic extract from the Bizerte locality at room temperature. The treated cells were subsequently seeded onto 96-well plates for 24 h. Following washing with PBS, cells were incubated with 3-[4,5-dimethylthiazol-2-yl]-2,5-diphenyl tetrazolium bromide (MTT) at 500 µg for 4 h. Formazan crystals resulting from MTT reduction were dissolved by adding a stopping solution and gently agitated for 30 min. The absorbance of the supernatant was then measured spectrophotometrically at 560 nm.

#### 4.9.2. Cell Proliferation Assay

U87 human glioblastoma cells were plated in 96-well plates (3 × 10^3^/well) in their complete medium and incubated for 24 h before being treated with *Mentha pulegium* L. methanol extract from the Bizerte locality. After incubation, the normal medium was replaced by a medium containing extract at 10 μg/mL and incubated for 48, 72, 96, and 120 h. The control cells were maintained in normal medium. At daily intervals, the wells were washed twice with PBS, and the attached cells were fixed with 3.7% formaldehyde. The fixed U87 cells were stained with a solution of 0.1% crystal violet and lysed with 1% SDS. Absorbance was then measured at 590 nm.

### 4.10. Statistical Analysis

The results were reported as the mean ± standard deviation. Analyses of variance (ANOVA) were performed by ANOVA procedures (SPSS 14.0 for Windows, SPSS Inc., Chicago, IL, USA). Significant differences between means were determined by Tukey’s post hoc tests; *p* values inferior to 0.05 were considered significant.

## Figures and Tables

**Table 1:** Averages of total phenolic and flavonoid contents and condensed tannin in the methanol extracts of *Mentha pulegium* L. leaves. Values are given as mean ± standard deviations (*p* < 0.05).

Locality	Total Phenol (GAE mg/g DW)	Flavonoids (RE mg/g DW)	Tannins (CE mg/g DW)
**Bizerte**	74.45 ± 0.01	28.87 ± 0.02	4.35 ± 0.02
**Kef**	57.43 ± 0.05	25.67 ± 0.1	1.57 ± 0.1

**Table 2:** Contents of *Mentha pulegium* L. polyphenolic compounds determined by HPLC (*p* < 0.05).

Identified Compound	Retention (min)	Content of Compounds (mg/g of Dry Weight)		Calibration Coefficient (r^2^)
		Bizerte	Kef	
caffeic acid **(1)**	10.50	8.55 ± 0.14	0.3 ± 0.01	0.999
eriocitrin **(2)**	12.50	25.3 ± 0.02	20.1 ± 0.10	0.999
isorhoifolin **(3)**	14.00	2.5 ± 0.1	1.2 ± 0.10	0.989
luteolin **(4)**	15.50	0.22 ± 0.02	0.2 ± 0.02	0.998
narirutin **(5)**	16.25	0.10 ± 0.01	0.09 ± 0.01	0.988
hesperidin **(6)**	17.50	0.75 ± 0.01	0.5 ± 0.08	0.993
rosmarinic acid **(7)**	18.50	12.65 ± 0.10	9.5 ± 0.08	0.994

Mean values of three independent experiments ± standard deviations. −r^2^: Calibration graphs were generated using five calibration solutions. All graphs were linear in the examined range (0.05–0.50 mg/mL) (*p* < 0.05).

**Table 3:** Antioxidant activities of methanolic extracts from Tunisian *Mentha pulegium* L. Values are given as mean ± SD (n = 3) (*p* < 0.05).

Locality	DPPH (IC_50_, μg/mL)	FRAP (μmol Fe^2+^/g)
**Bizerte**	16.31 ± 0.94	570.08 ± 0.85
**Kef**	19.08 ± 0.83	481.01 ± 0.96

**Table 4:** Antibacterial activity of methanolic extracts of *Mentha pulegium* L. leaves determined by the disc diffusion method (*p* < 0.05).

	Inhibition Zone Diameters (mm)						
	**Bizerte/Concentrations (mg/mL)**			**Kef/Concentrations (mg/mL)**			**Gentamicin**
	0.1	0.5	2	0.1	0.5	2	(15 µg/disc)
**Gram-negative Bacteria**							
*Klebsiella pneumoniae*	11 ± 0.37	15 ± 0.33	21 ± 0.33	9 ± 0.3	13 ± 0.22	19 ± 0.1	+
*Escherichia coli*	13 ± 0.0	19 ± 0.35	25 ± 0.22	8 ± 0.1	17 ± 0.2	22 ± 0.5	24 ± 0.0
*Shigella boydii*	12 ± 0.35	18 ± 0.3	24 ± 0.35	9 ± 0.22	15 ± 0.5	22 ± 0.3	20 ± 0.0
*Vibrio cholerae*	11 ± 0.25	19 ± 0.22	23 ± 0.25	9 ± 0.33	16 ± 0.1	20 ± 0.2	21 ± 0.0
**Gram-positive Bacteria**							
*Streptococcus aureus*	21 ± 0.15	25 ± 0.3	33 ± 0.22	19 ± 0.1	22 ± 0.22	30 ± 0.1	27 ± 0.0
*Bacillus subtilis*	17 ± 0.22	24 ± 0.1	31 ± 0.33	12 ± 0.2	22 ± 0.22	29 ± 0.3	25 ± 0.0
*Clostridium tetani*	20 ± 0.5	25 ± 0.3	32 ± 0.15	13 ± 0.3	22 ± 0.33	22 ± 0.0	23 ± 0.0
Enterococcus	15 ± 0.5	24 ± 0.2	30 ± 0.3	12 ± 0.2	20 ± 0.3	26 ± 0.33	+

+: growth.

**Table 5:** Results of minimum inhibitory concentration (MIC) and minimum bactericidal concentration (MBC) of methanolic extracts of *Mentha pulegium* L.

	MIC (µg/mL)		MBC (µg/mL)	
	Bizerte	Kef	Bizerte	Kef
**Gram-negative Bacteria**				
*Klebsiella pneumoniae*	80	160	160	320
*Escherichia coli*	80	160	160	320
*Shigella boydii*	40	80	80	160
*Vibrio cholerae*	80	160	160	320
**Gram-positive Bacteria**				
*Streptococcus aureus*	40	160	80	320
*Bacillus subtilis*	40	80	80	160
*Clostridium tetani*	40	80	80	160
*Enterococcus*	80	160	160	320

## Data Availability

Not applicable.

## Sample Availability

Samples of the compounds are available from the authors.

## References

[1] Attiya J., Bin G., Bilal H.A., Zabta K.S., Tariq M. (2012). Phylogenetics of selected Menthaspecies on the basis of rps8, rps11 and rps14 chloroplast genes. J. Med. Plants Res..

[2] Brahmi F., Hauchard D., Guendouze N., Madani K., Kamagaju L., Stévigny C., Chibane M., Duez P. (2015). Phenolic composition, in vitro antioxidant effects and tyrosinase inhibitory activity of three Algerian Mentha species: *M. spicata* (L.), *M. pulegium* (L.) and *M. rotundifolia* (L.) Huds (Lamiaceae). Ind. Crop. Prod..

[3] Mkaddem M., Boussaid N.M., Ben Fadhel N. (2011). Variability of Volatiles in Tunisian *Mentha pulegium* L. (Lamiaceae). J. Essent. Oil Res..

[4] Sutour S., Bradesi P., Casanova J., Tomi F. (2010). Composition and chemical variability of Mentha suaveolens ssp. suaveolens and M. suaveolens ssp. insularis from Corsica. Chem. Biodivers..

[5] Ghazghazi H., Chedia A., Weslati M., Trakhna F., Houssine S., Abderrazak M., Brahim H. (2013). Chemical composition and in vitro antimicrobial activites of menthe pulegium leaves extracts against food borne pathogens. J. Food Saf..

[6] Mahendran G., Rahman L.U. (2020). Ethnomedicinal, phytochemical and pharmacological updates on Peppermint *Mentha piperita* L.. Phytother Res..

[7] Ben H.J., Yahia I., Zaouali Y., Ciavatta M.L., Ligresti A., Jaouadi R., Boussaid M., Cutignano A. (2019). Polyphenolic Profiling, Quantitative Assessment and Biological Activities of Tunisian Native *Mentha rotundifolia* (L.) Huds. Molecules.

[8] Khan M., Saeed Abdullah M.M., Mousa A.A., Alkhathlan H.Z. (2016). Chemical composition of vegetative parts and flowers essential oils of wild Anvillea garcinii grown in Saudi Arabia. Rec. Nat. Prod..

[9] Khan M., Mahmood A., Alkhathlan H.Z. (2016). Characterization of leaves and flowers volatile constituents of *Lantana camara* growing in central region of Saudi Arabia. Arab. J. Chem..

[10] Khan M., Garg A., Srivastava S.K., Darokar M.P. (2012). A cytotoxic agent from Strychnos nux-vomica and biological evaluation of its modified analogues. Med. Chem. Res..

[11] Alharbi N.K., Naghmouchi S., Al-Zaban M. (2021). Evaluation of Antimicrobial Potential and Comparison of HPLC Composition, Secondary Metabolites Count, and Antioxidant Activity of *Mentha rotundifolia* and *Mentha pulegium* Extracts. Evid. Based Complement Alternat. Med..

[12] Sebai E., Serairi R., Saratsi K., Abidi A., Sendi N., Darghouth M.A., Wilson M.S., Sotiraki S., Akkari H. (2020). Hydro-Ethanolic Extract of Mentha pulegium Exhibit Anthelmintic and Antioxidant Proprieties In Vitro and In Vivo. Acta Parasitol..

[13] Sebai E., Abidi A., Serairi R., Marzouki M., Saratsi K., Darghouth M.A., Sotiraki S., Akkari H. (2021). Essential oil of Mentha pulegium induces anthelmintic effects and reduces parasite-associated oxidative stress in rodent model. Exp. Parasitol..

[14] Manosroi J., Dhumtanom P., Manosroi A. (2005). Anti-proliferative activity of essential oil extracted from Thai medicinal plants on KB and P388 cell lines. Cancer Lett..

[15] Shirazi F.H., Ahmadi N., Kamalinejad M. (2004). Evaluation of northern Iran *Mentha Pulegium,* L. cytotoxicity Journal. Pharm. Sci..

[16] Hajighasemi F., Hashemi V., Khoshzaban F. (2011). Cytotoxic effect of *Mentha spicata* aqueous extract on cancerous cell lines in vitro. J. Med. Plants Res..

[17] Conforti F., Ioele G., Statti G.A., Marrelli M., Ragno G., Menichini F. (2008). Antiproliferative activity against human tumor cell lines and toxicity test on Mediterranean dietary plants. Food Chem. Toxicol..

[18] Hajlaoui H., Snoussi M., Ben Jannet H., Mighri Z., Bakhrouf A. (2008). Comparison of chemical composition and antimicrobial activities of *Mentha longifolia* L. ssp. longifolia essential oil from two Tunisian localities (Gabes and Sidi Bouzid). Ann. Microbiol..

[19] Karray N., Ksouri R., Falah H., Rabhi M., Abduljaleel C., Grignon C., Lachaal M. (2010). Effects of environement and development stage on phenolic contenant and antioxidant activities of *Mentha Pulegium* L.. J. Food Biochem..

[20] Padmini E., Valarmathi A., Usha R.M. (2010). Comparative analysis of chemical composition and antibacterial activities of *Mentha spicata* and Camellia sinensis. Asian J. Exp. Biol. Sci..

[21] Sroka Z., Fecka I., Cisowski W. (2005). Antiradical and Anti-H_2_O_2_ Properties of Polyphenolic Compounds from an Aqueous Peppermint Extract. Z. Naturforsch..

[22] Bodalska A., Kowalczyk A., Włodarczyk M., Fecka I. (2019). Analysis of Polyphenolic Composition of a Herbal Medicinal Product-Peppermint Tincture. Molecules.

[23] Riahi L., Elferchichi M., Ghazghazi H., Jebali J., Ziadi S., Aouadhi C., Chograni H., Zaouali Y., Zoghlami N., Mliki M. (2013). Phytochemistry, antioxidant and antimicrobial activities of the essential oils of *Mentha rotundifolia* L. in Tunisia. Ind. Crops. Prod..

[24] Kokkini S., Papageorgiou V.P. (1988). Constituents of Essential Oils from Mentha X rotundifolia Growing Wild in Greece. Planta Med..

[25] El Arch M., Satrani M., Farah A., Bennani L., Boriky L., Fechtal M., Mohamed B., Talbi M. (2003). Composition chimique et activités antimicrobienne et insecticide de l’huile essentielle de Mentha rotundifolia du Maroc. Acta Bot. Gallica..

[26] Brada M., Bezzina M., Marlier M., Carlier A., Lognay G. (2007). Variabilité de la composition chimique des huiles essentielles de *Mentha rotundifolia* du Nord de l’Algérie. J. Biotechnol. Agron. Soc. Environ..

[27] Sanchez-Moreno C. (2002). Methods used to evaluate the free radical scavenging activity in foods and biological systems. Food Sci. Technol. Int..

[28] Nakamura Y., Ohto Y., Murakami A., Ohigashi H. (1998). Superoxide scavenging activity of rosmarinic acid from Perilla frutescens Britton var. acuta f. viridis. J. Agric. Food Chem..

[29] Fuhrman B., Volkova N., Rosenblat M., Aviram M. (2000). Lycopene synergistically inhibits LDL oxidation in combination with vitamin E, glabridin, rosmarinic acid, carnosic acid, or garlic acid. Antioxid. Redox. Signal..

[30] Hajlaoui H., Trabelsi N., Noumi E., Snoussi M., Fallah H., Ksouri R., Bakhrouf A. (2010). Biological activities of the essential oils and methanol extract of tow culti-vated mint species (*Mentha longifolia* and *Mentha pulegium*) used in the Tunisian folkloric medicine. World J. Microbiol. Biotechnol..

[31] Zhang Y.M., White S.W., Rock C.O. (2006). Inhibiting bacterial fatty acid synthesis. J. Biol. Chem..

[32] Kelmanson J.E., Jäger A.K., van Staden J. (2000). Zulu medicinal plants with antibacterial activity. J. Ethnopharmacol..

[33] Masika P.J., Afolayan A.J. (2002). Antimicrobial activity of some plants used for the treatment of livestock disease in the Eastern Cape, South Africa. J. Ethnopharmacol..

[34] Marzouk B., Ben Had J., Fredj M., Chraief I., Mastouri M., Boukef K., Marzouk Z. (2008). Chemical composition and antimicrobial activity of essential oils from Tunisian *Mentha pulegium* L.. J. Food Agric. Environ..

[35] Mahboubi M., Haghi G. (2008). Antimicrobial activity and chemical composition of *Mentha pulegium* L. essential oil. J. Ethnopharmacol..

[36] Boukhebti H., Chaker A.N., Belhadj H., Sahli F., Messaoud R., Laouer H., Daoud Harzallah D. (2011). Chemical composition and antibacterial activity of *Mentha pulegium* L. and *Mentha spicata L*. essential oils. Der. Pharm. Lett..

[37] Nikaido H., Vaara M. (1985). Molecular basis of bacterial outer membrane permeability. Microbiol. Rev..

[38] Vaara M. (1992). Agents that increase the permeability of the outer membrane. MicroBiol. Rev..

[39] Gulluce M., Sahin F., Sokmen M., Ozer H., Daferera D., Sokmen A., Polissiou M., Adiguzel A., Ozkan H. (2007). Antimicrobial and antioxidant properties of the essential oils and methanol extract from *Mentha longifolia* L. ssp. Longifolia. Food Chem..

[40] Brantner A., Males Z., Pepeljnjak S., Antolić A. (1996). Antimicrobial activity of Paliurus spina-christi Mill. (Christ’s thorn). J. Ethnopharmacol..

[41] Mossmann T. (1983). Rapid calorimetric assay for cellular growth and survival: Application to proliferation and cytotoxicity assays. J. Immunol Met..

[42] Hossan M.S., Rahman S., Anwarul Bashar A.B.M., Jahan R., Al-Nahain A., Mohammed R. (2014). Rosmarinic acid: A review of its anticancer action. World J. Pharm. Pharm. Sci..

[43] Majumdar D., Jung K.H., Zhang H., Nannapaneni S., Wang X., Amin A.R., Chen Z., Chen Z.G., Shin D.M. (2014). Luteolin nanoparticle in chemoprevention: In vitro and in vivo anticancer activity. Cancer Prev. Res..

[44] Ozturk G., Ginis Z., Akyol S., Erden G., Gurel A., Akyol O. (2012). The anticancer mechanism of caffeic acid phenethyl ester (CAPE): Review of melanomas, lung and prostate cancers. Eur. Rev. Med. Pharm. Sci..

[45] Mau J.L., Chao G.R., Wu K.T. (2001). Antioxidant properties of methanolic extracts from several ear mushrooms. J. Agric. Food Chem..

[46] Rice-Evans C.A., Miller N.J., Paganga G. (1996). Structure-antioxidant activity relationships of flavonoids and phenolic acids. Free Radic. Biol. Med..

[47] Sun B., Ricardo-da-Silva J.M., Spranger I. (1998). Critical factors of vanillin assay for catechin and proanthocyanidins. J. Agric. Food Chem..

[48] Slinkard K., Singletont V.L. (1977). Total phenol analysis: Automation and comparison with manual methods. Am. J. Enol. Vitic..

[49] Hatano T., Kagawa H., Yasuhara T., Okuda T. (1988). Two new flavonoids and other constituents in licorice root: Their relative astringency and radical scavenging effects. Chem. Pharm. Bull..

[50] Benzie I.F., Strain J.J. (1996). The ferric reducing ability of plasma (FRAP) as a measure of “antioxidant power”: The FRAP assay. Anal. Biochem..

[51] Aouadhi C., Simonin H., Maaroufi A., Mejri S. (2013). Optimization of nutrient-induced germination of Bacillus sporothermodurans spores using response surface methodology. Food Microbiol..

